# Intraoperative ultrasound in recurrent gliomas surgery: Impact on residual tumor volume and patient outcomes

**DOI:** 10.3389/fonc.2023.1161496

**Published:** 2023-03-23

**Authors:** Meiyao Wang, Jin Yu, Jibo Zhang, Zhiyong Pan, Jincao Chen

**Affiliations:** ^1^ Department of Neurosurgery, Zhongnan Hospital of Wuhan University, Wuhan, China; ^2^ Department of Neurology, University Hospital Zurich, Zurich, Switzerland

**Keywords:** recurrent gliomas, surgical resection, intraoperative ultrasound, localization, guidance, postoperative residual, patient outcomes

## Abstract

**Background:**

Reoperation may be beneficial for patients with recurrent gliomas. Minimizing the residual tumor volume (RTV) while ensuring the functionality of relevant structures is the goal of the reoperation of recurrent gliomas. Intraoperative ultrasound (IoUS) may be helpful for intraoperative tumor localization, intraoperative real-time imaging to guide surgical resection, and postoperative evaluation of the RTV in the reoperation for recurrent gliomas.

**Objective:**

To assess the effect of real-time ioUS on minimizing RTV in recurrent glioma surgery compared to Non-ioUS.

**Methods:**

We retrospectively analyzed the data from 92 patients who had recurrent glioma surgical resection: 45 were resected with ioUS guidance and 47 were resected without ioUS guidance. RTV, Karnofsky Performance Status (KPS) at 6 months after the operation, the number of recurrent patients, and the time to recurrence were evaluated.

**Results:**

The average RTV in the ioUS group was significantly less than the Non-ioUS group (0.27 cm^3^ vs. 1.33 cm^3^, p = 0.0004). Patients in the ioUS group tended to have higher KPS scores at 6 months of follow-up after the operation than those in the Non-ioUS group (70.00 vs. 60.00, p = 0.0185). More patients in the Non-ioUS group experienced a recurrence than in the ioUS group (43 (91.49%) vs. 32 (71.11%), p = 0.0118). The ioUS group had a longer mean time to recurrence than the Non-ioUS group (7.9 vs. 6.3 months, p = 0.0013).

**Conclusion:**

The use of ioUS-based real-time for resection of recurrent gliomas has been beneficial in terms of both RTV and postoperative outcomes, compared to the Non-ioUS group.

## Introduction

In the case of glioma treatment, recurrence is a question of time (1–4). There is currently no agreement on the protocol for treating recurrent gliomas, reoperation may be beneficial for the development of the disease (5–7). Reoperation of recurrent gliomas is more challenging for surgeons because of scar tissue, distorted anatomical markers, more diffuse tumor boundaries, and scattered multifocal lesions left by previous surgery and adjuvant treatment. However, the goal of reoperation remains to remove the recurrent tumor as completely as possible to minimize the residual tumor volume (RTV) while trying to preserve the functionality of relevant structures, which is known to improve patient survival and surgical outcomes (8–10).

In recent years, intraoperative ultrasound (ioUS) in neurosurgical brain tumor surgery is helpful for intraoperative tumor localization, intraoperative real-time imaging to guide surgical resection, and postoperative evaluation of the RTV (11–13), which is not affected by brain shift due to a reduction in cerebrospinal fluid after craniotomy and the change of tumor location during surgical operation (14, 15). Meanwhile, ioUS demonstrates several important advantages, such as low cost, rapid repeatability, real-time scanning of the surgical field, portability, and user-friendliness (16–19).

Although the advantages of ioUS in the first surgery for gliomas have been widely researched and reported (18, 20), not much is known regarding reoperation for recurrent gliomas. Therefore, we assessed the patients who had undergone recurrent glioma reoperation using ioUS to find out if the application of ioUS has an impact on the RTV, and understand how ioUS-guided surgery can impact RTV and early postoperative neurological outcomes in patients with recurrent glioma.

## Methods

### Patients’ characteristics

We searched the computerized medical records at our institution for appropriate individuals. Inclusion criteria were age < 80 years, a KPS score > 60, a histopathological diagnosis of Astrocytoma, IDH-mutant, WHO grades 3–4, Glioblastoma, IDH-wildtype, WHO grade 4, or oligodendroglioma, IDH-mutant and 1p/19q-codeleted, WHO grades 2–3 that after a first surgery (The pathology was determined by a senior neuropathologist in all cases, and the grading criteria were based on the gliomas in the 2021 edition of the World Health Organization (WHO) classification of central nervous system tumors), and a feasible gross-total resection of recurrent tumor according to preoperative MRI. Exclusion criteria were age > 80 years, a low KPS score (≤ 60), and poor health in general. A total of 92 patients with recurrent glioma underwent surgical resection, and their data were assessed retrospectively. Surgeries were performed, between January 2016 and October 2022, by neurosurgeons with at least ten years of surgical experience who are board-certified, at the Department of Neurosurgery, Zhongnan Hospital of Wuhan University, Wuhan, Hubei, China. The glioma’s grade was determined *via* a histopathological diagnostic. Patients were subgrouped according to the use of intraoperative ultrasound (ioUS): of those patients, a total of 45 underwent surgery with the assistance of real-time intraoperative ultrasound (ioUS group); the remaining 47 underwent surgery without such assistance (Non-ioUS group). Patients with tumors in eloquent areas, such as the Broca or Wernicke area, motor cortex, thalamus, and basal ganglia, underwent surgery while awake using techniques such as motor evoked potentials, cortical and subcortical stimulation, and sensory evoked potentials. Patients with non-eloquent tumors underwent surgery while under general anesthesia. Preoperative tumor volume was evaluated by preoperative magnetic resonance imaging (MRI) scans and preoperative Karnofsky Performance Status (KPS) was evaluated. In accordance with the Response Assessment in Neuro-Oncology criteria, progression-free survival (PFS) following a first operation was determined from the date of the first surgery to the date of documented evidence of disease progression. The ioUS group and the Non-ioUS group were compared in parallel. The study was approved by the institutional review board, all patients were fully informed about the surgical technique, and signed consent was collected.

### MRI and CT assessment

All patients performed preoperative MRI and computed tomography (CT) for surgical planning and the determination of preoperative tumor volume. The postoperative MRI and CT for the evaluation of tumor residual. MRI scans were conducted on a 3.0 T MRI scanner uMR790 (United Imaging Healthcare). A GE discovery 750HD scanner (GE Medical Systems, Milwaukee, WI, USA) was used for CT scans. Tumor volumes were evaluated by manual segmentation using the ITK-SNAP software. After case discussion among the neurosurgeons, neurooncologists, and radiation oncologists, patients were typically recommended a second operation for recurrent tumors. Typically, recurrent tumors were discovered on routine postoperative MRI scans that were carried out three months after the first surgery or if symptoms like worsening headaches, muscle weakness, or other deficiencies appeared. Likewise, the surgeon, radiation oncologist, medical oncologist, and patients themselves decided on the specific adjuvant radiation and chemotherapy treatments to utilize.

### IoUS assessment

**Figure 1:**
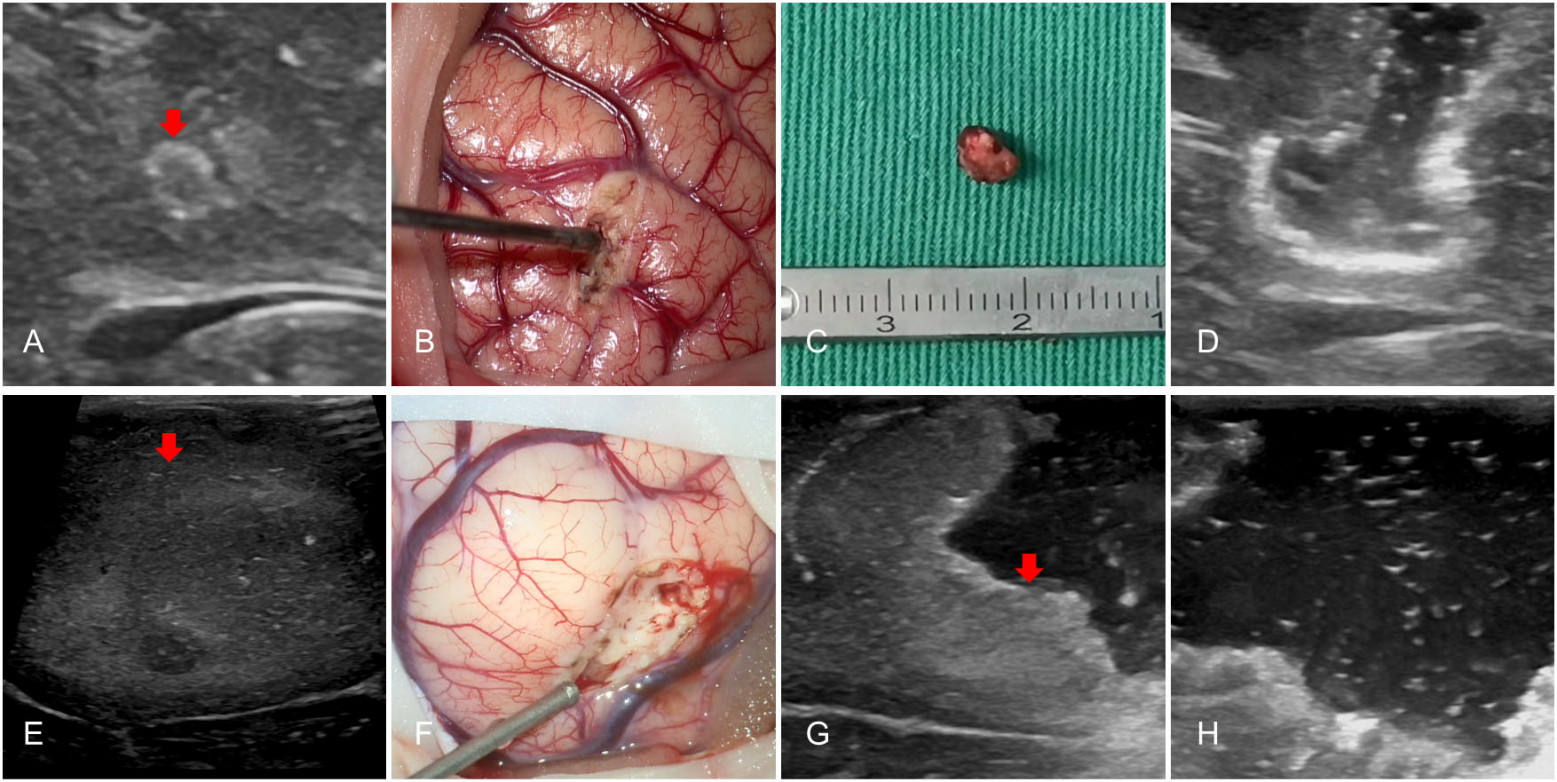
IoUS done prior to opening the dura revealed one little hyperechoic signal in the surgical area (**A, E**, red arrow). A second ioUS was carried out after the dura was opened to locate the tumor on the surface of the brain **(B, F)**. The tumor was resected with the real-time repeated ioUS guidance **(C)**. A hyperechogenic lesion presumed to be a tumor remnant was discernible when the doctor used the US to evaluate the surgical field near the anticipated end of the resection (**G**, red arrow). The doctor used the US to evaluate the surgical field after the excision and found no tumor remnants **(D, H)**.

All patients (ioUS and non-ioUS control groups) were operated on using preoperative MRI and CT. In the Non-ioUS control group, microsurgical tumor resections were completed according to preoperative MRI and CT, the eloquent or non-eloquent tumor location, and the surgeon’s experience. In the ioUS group, except for the preoperative MRI and CT, we used a US system (GE LOGIQ E, USA) to obtain the US images. IoUS was performed by doctors with expertise and training in the US. The probe utilized is a variable band linear transducer with a bandwidth of 4.5 to 14.0 MHz (GE L8-18i-RS, USA), a sterile cover is used for the execution of the ioUS scan and after the bone flap is removed, the first ioUS is performed before accessing the dura to correctly identify the lesion and surrounding structures (**Figures 1A–E**). A second ioUS was carried out after the dura was opened to find the tumor on the surface of the brain (**Figures 1B–F**). IoUS can be used numerous times during surgery to provide real-time guidance in locating the lesion for excision. (**Figure 1C**). To determine whether there was any remaining tumor tissue after the microsurgical removal of all visible tumor tissue, ioUS was used. If there was no residual tumor (**Figure 1D**), the resection was completed. Further excision was carried out when a tumor remnant was seen on ioUS images (**Figure 1G**). Repeated ioUS was performed to confirm complete resection (**Figure 1H**). Surgery was completed according to the preoperative MRI and CT, the ioUS observations, the eloquent or non-eloquent tumor area, and the surgeon’s assessment.

### Postoperative patients’ outcomes

All patients underwent an early postoperative MRI 48 hours following surgery, which was compared to preoperative MRI images. When comparing the postoperative MRI scans to the preoperative MRI images, complete resection was defined as the absence of solid tumor remains. Utilizing a specific instrument from the operating station, residual tumor volume was assessed using manual segmentation and a volume rendering approach. A third-year neurosurgery resident assessed tumor volumes, which were then confirmed by a board-certified neurosurgeon. Preoperative and postoperative KPS scores were assessed. Postoperative patients were followed up for one year. The KPS at 6 months after the operation, whether relapse, and time to recurrence were recorded.

### Statistical analysis

The Student t-test, Mann-Whitney U test, Chi-square test, and Fisher’s exact test were used in the statistical analysis. GraphPad Prism 8.0 (GraphPad Software, San Diego, California) was used to conduct the statistical analysis. P<0.05 was regarded as statistically significant.

## Results

### Patients’ characteristics

A total of 92 patients were involved in the study (mean age of 38.71 years, 45 males and 47 females). All patients were diagnosed with recurrent gliomas, 8 of them were WHO grade 2, 21 were WHO grade 3 and 63 were WHO grade 4. 45 patients were operated on with the use of a real-time ioUS, and 47 of them were operated on without the use of a real-time ioUS. There were 25 patients with tumors in eloquent regions and 67 individuals with tumors in non-eloquent areas. The mean preoperative tumor volume is 4.00cm^3^ and the median preoperative KPS is 80 (interquartile range (IQR) is 70-80). The mean PFS after 1st surgery was 26.28 (range from 1 to 108). The cohort’s detailed clinical characteristics are summarized in **Table 1**.

**Table 1:** Preoperative overall characteristics of patients with recurrent gliomas.

Patients’ characteristics	Overall (n = 92)
Age (Mean ± SD)	38.71 ± 13.28
Gender	
Male (%)	45 (48.9)
Female (%)	47 (51.09)
Grade of recurrent gliomas	
WHO grade 2 (%)	8 (8.69)
WHO grade 3 (%)	21 (22.83)
WHO grade 4 (%)	63 (68.48)
ioUS (%)	45 (48.91)
Non-ioUS (%)	47 (51.09)
Localization	
Eloquent (%)	25 (27.17)
Non-eloquent (%)	67 (72.83)
Tumor volume cm^3^ (Mean ± SD)	4.00 ± 2.22
Preoperative KPS (median [IQR])	80.00 [70.00, 80.00]
PFS after 1st surgery (months, (Mean [Range]))	26.28 [1 – 108]

SD, standard deviation; WHO, World Health Organization; ioUS, intraoperative Ultrasound; KPS, Karnofsky Performance Status; IQR, Interquartile Range; PFS, Progression-Free Survival.

The 2 patient groups (ioUS and Non-ioUS) were comparable for sex, age, grade of recurrent gliomas, tumor localization, preoperative tumor volume, and preoperative KPS and PFS after 1st surgery (no significant difference, p > 0.05, **Table 2**).

**Table 2:** Comparison between patients undergoing ioUS and patients not undergoing ioUS localization and guided surgical resection of recurrent gliomas.

Patients’ characteristics	ioUS (n = 45)	Non-ioUS (n = 47)	P	Test
Age (years, Mean ± SD)	34.78 ± 14.95	39.19 ± 11.19	0.1113	Student t
Gender			0.4139	Fisher’s exact
Male (%)	20 (44.44)	25 (53.19)		
Female (%)	25 (55.56)	22 (46.81)		
Grade of recurrent gliomas			0.7235	Chi-square
WHO grade 2 (%)	5 (11.11)	3 (6.38)		
WHO grade 3 (%)	10 (22.22)	11 (23.40)		
WHO grade 4 (%)	30 (66.67)	33 (70.21)		
Localization			0.2435	Fisher’s exact
Eloquent (%)	15 (33.33)	10 (21.28)		
Non-eloquent (%)	30 (66.67)	37 (78.72)		
Tumor volume (cm^3^, Mean ± SD)	4.10 ± 2.44	3.90 ± 2.01	0.6627	Student t
Preoperative KPS (median [IQR])	80.00 [70.00, 90.00]	80.00 [70.00, 80.00]	0.6450	Non-norm (Mann Whitney)
PFS after 1st surgery (months, (Mean [Range]))	24.04 [1 – 96]	26.28 [3 – 108]	0.1052	Non-norm (Mann Whitney)

SD, standard deviation; WHO, World Health Organization; ioUS, intraoperative Ultrasound; KPS, Karnofsky Performance Status; IQR, Interquartile Range; PFS, Progression-Free Survival.

### Postoperative patients’ outcomes

A postsurgical residual tumor volume (RTV) is a significant indicator of poor patient outcomes in glioma. RTV was discovered at the end of surgery with the US in 35/45 (77.78%) of the ioUS group. Surgeons continued the procedure in 20 of the 35 patients until full resection was established by the US. The discovered residual tumor could not be completely resected in the remaining 15 individuals due to its proximity to important structures. After surgery, MRI in all 45 individuals revealed the existence of a residual tumor, with an average postoperative volume of 0.27 cm^3^ (range 0.00 - 1.00 cm^3^). The postoperative MRI revealed the full eradication of the tumor mass in 10 of the 47 (21.28%) patients in the Non-ioUS control group. In all of these 47 cases, the mean residual volume was 1.33 cm^3^ on average (range 0.00 – 4.88 cm^3^). There was a significant difference between the ioUS and the non-ioUS groups concerning residual tumor volume. Resection tended to be more complete in the ioUS group (ioUS vs. Non-ioUS, mean RTV 0.27 vs. 1.33, p = 0.0004). In the ioUS group, the KPS score decreased from a preoperative median of 80 (IQR: 70 - 90) to a postoperative median of 70 (IQR: 65 - 80). At the follow-up 6 months after the operation, the mean KPS score was 70 (IQR: 60 - 70). In the Non-ioUS control group, the median preoperative KPS score was 80 (IQR: 70 - 80), whereas the median postoperative KPS score was 70 (IQR: 70 - 80). But at the follow-up 6 months after the operation, the mean KPS score was 60 (IQR: 60 - 70). Although there was no difference in postoperative KPS between the ioUS and Non-ioUS groups, there was a significant difference between ioUS and Non-ioUS groups concerning the KPS scores at 6 months after the operation. Those in the ioUS group tended to have higher KPS scores at 6 months of follow-up after the operation than patients in the Non-ioUS group (p = 0.0185). More patients had a recurrence in the Non-ioUS group than patients in the ioUS group ((ioUS vs. Non-ioUS, 43 (91.49%) vs. 32 (71.11%), p = 0.0118). The ioUS group had a longer mean time to recurrence than the Non-ioUS group (7.9 months (range 4 – 12 months) vs. 6.3 months (range 2 – 11 months, p = 0.0013) (**Table 3**).

**Table 3:** Outcomes of patients who underwent a second surgery for recurrent gliomas according to ioUS versus non-ioUS.

Results	ioUS (n = 45)	Non-ioUS (n = 47)	P	Test
RTV (cm^3^, Mean [Range])	0.27 [0.00 – 1.00]	1.33 [0.00 – 4.88]	**0.0004**	Non-norm (Mann Whitney)
Postoperative KPS (median [IQR])	70.00 [65.00, 80.00]	70.00 [70.00, 80.00]	0.3759	Non-norm (Mann Whitney)
KPS at 6 months after the operation (median [IQR])	70.00 [60.00, 70.00]	60.00 [60.00, 70.00]	**0.0185**	Non-norm (Mann Whitney)
Number of patients			**0.0118**	Chi-square
Recurrence	32 (71.11%)	43 (91.49%)		
No recurrence	13 (28.89%)	4 (8.51%)		
Time to recurrence (months, (Mean [Range]))	7.91 [4-12]	6.30 [2-11]	**0.0013**	Non-norm (Mann Whitney)

RTV, Residual Tumor Volume; KPS, Karnofsky Performance Status; IQR, Interquartile Range.

Statistically significant differences (*p*-value <0 .05) are highlighted in bold.

To analyze the reason why intraoperative ultrasound can reduce RTV, we carefully compared the preoperative and postoperative CT, MRI, US, and pathological results of the patients, number of tumors in three patients is listed in **Table 4**. Details are as follows.

**Table 4:** Number of tumors in representing patients on preoperative CT, MRI, ioUS, postoperative US, postoperative CT and MRI.

Patients	preoperative CT	preoperative MRI	ioUS	postoperative US	postoperative CT	Postoperative MRI
M.X.Y.	–	1	3	0	0	–
Z.K.Y.	4	1	4	0	0	0
J.L.X	–	1	2	0	0	0

CT, Computed Tomography; MRI, Magnetic Resonance Imaging; ioUS, intraoperative Ultrasound; US, Ultrasound.

#### Patient 1

**Figure 2:**
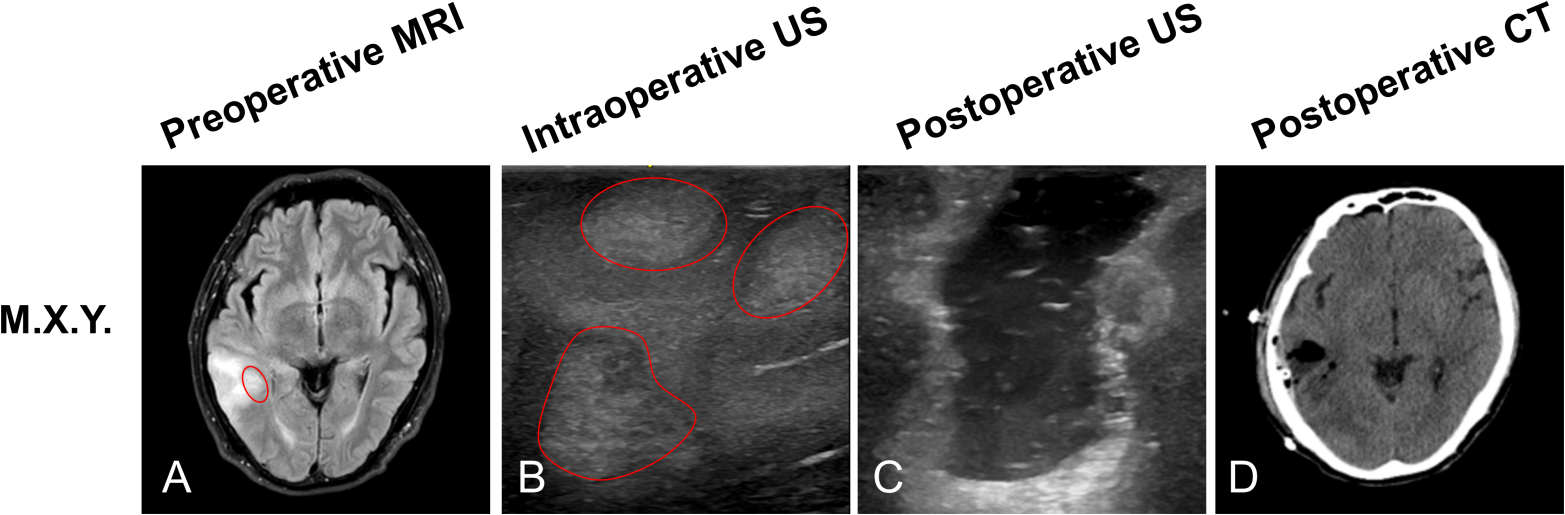
Illustrating case 1: M.X.Y, a 57-year-old male patient, MRI sequences showed one tumor located at the right temporal (**A**, red circle). Prior to opening the dura, ioUS revealed three tumors (three hyperechoic signals) in the operative field (**B**, red circles). The doctor used the ioUS to evaluate the surgical field at the ending of the resection and found no tumor remnants **(C)**. The postoperative CT scan reveals no signs of the tumor **(D)**. Histopathology indicated that the patient had recurrent Glioblastoma WHO grade 4.

M.X.Y, a 57-year-old male patient, preoperative MRI sequences showed one tumor located at the right temporal (**Figure 2A**, red arrow). Three hyperechoic signals (three tumors) were detected by ioUS before the dura was opened in the operative field (**Figure 2B**, red arrow). Surgeons removed the three lesions separately according to intraoperative ultrasound guidance. The three lesions were loaded into specimen bags for pathological examination. Histopathology revealed that all three lesions were recurrent glioblastoma WHO grade 4. When the surgeon used ioUS to check the operative field after the resection, no tumor remnants were seen (**Figure 2C**). The postoperative CT scan reveals no signs of the tumor (**Figure 2D**).

#### Patient 2

**Figure 3:**
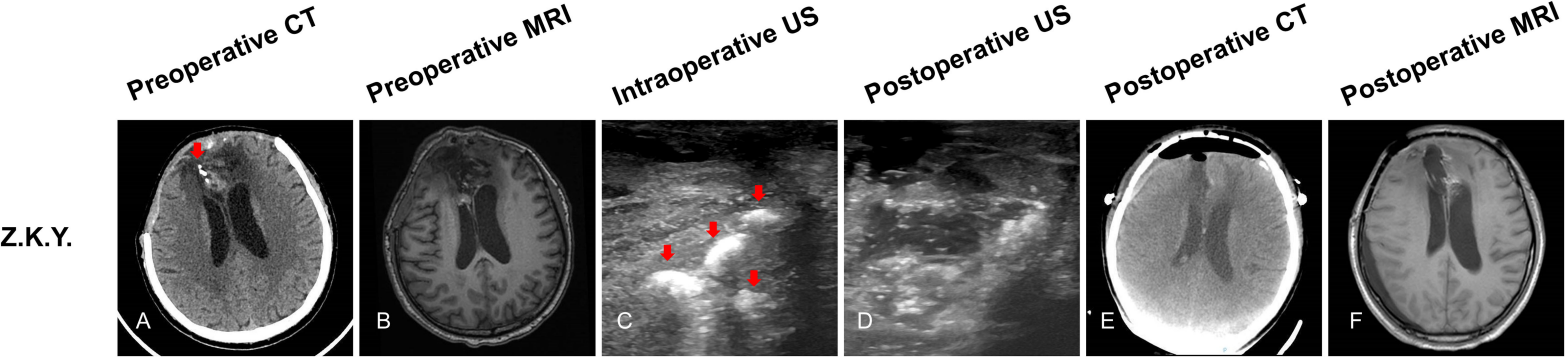
Illustrating case 2: Z.K.Y, a 50-year-old male patient, CT scan showed four high density (four tumors) located at the right frontal lobe (**A**, red arrow). MRI (T1WI) sequences showed only one tumor **(B)**. Before opening the dura, ioUS revealed four hyperechoic signals (four tumors) in the operative field (**C**, red arrow). When the doctor used the ioUS to check the surgical field after the resection, no tumor remnants were found **(D)**. The postoperative CT scan shows no tumor remnant **(E)**. The postoperative MRI (T1WI) scan reveals no signs of the tumor **(F)**. Recurrent WHO grade 2 oligodendroglioma was discovered by histopathology.

Z.K.Y, a 50-year-old male patient, preoperative CT scan that showed four high density (four tumors) located at the right frontal lobe (**Figure 3A**, red arrow). preoperative MRI sequences showed only one tumor (**Figure 3B**). Before opening the dura, ioUS revealed four hyperechoic signals (four tumors) in the operative field (**Figure 3C**, red arrow). Surgeons removed the four lesions separately according to intraoperative ultrasound guidance. Histopathology revealed recurrent oligodendroglioma WHO grade 2. When the surgeon used ioUS to check the operative field after the resection, no tumor remnants were seen (**Figure 3D**). The postoperative CT scan reveals no signs of the tumor (**Figure 3E**). No tumor remains are visible on the postoperative MRI scan (**Figure 3F**).

#### Patient 3

**Figure 4:**
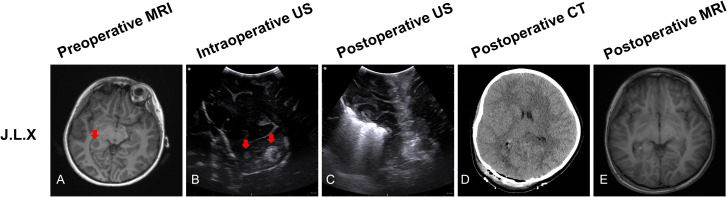
Illustrating case 3: J.L.X, a 7-year-old girl, MRI (T1WI) sequences showed one tumor located at the right periventricular (**A**, red arrow). Before opening the dura, IoUS found two hyperechoic signals (two tumors) in the operative field (**B**, red arrow). After the resection, the doctor used the ioUS to check the surgical field, and no tumor remnants were found **(C)**. The postoperative CT scan reveals no tumor remnants **(D)**. No tumor remains are visible on the postoperative MRI (T1WI) scan **(E)**. Histopathology indicated recurrent WHO grade 3 glioma.

J.L.X, a 7-year-old girl, preoperative MRI sequences showed one tumor located at the right periventricular (**Figure 4A**, red arrow). Prior to opening the dura, ioUS revealed two tumors (two hyperechoic signals) in the operative field (**Figure 4B**, red arrow). Surgeons removed the two lesions separately according to intraoperative ultrasound guidance. Histopathology revealed that all two lesions were recurrent glioma WHO grade 3. The doctor used ioUS to evaluate the surgical field after the excision and found no tumor remnants (**Figure 4C**). The postoperative CT scan reveals no signs of the tumor (**Figure 4D**). No tumor remains are visible on the postoperative MRI scan (**Figure 4E**).

## Discussion

There is no universal agreement on the optimal treatment strategy for glioma recurrence. Even though the consistent effect was unclear, Kirkpatrick and Sampson investigated several therapy strategies and identified re-operation as a beneficial treatment choice (21). The survival rate after two resections has increased, according to recent surgical experience. According to Montemurro et al., patients who underwent their first and second gross-total resections for recurrent glioblastoma experienced an improvement in overall survival (HR = 0.195, 95% CI 0.091-0.419; p < 0.0001) (22). Another study found that the reoperation group outlived the non-reoperation group by 16.4 and 10.5 months in overall survival and 3.5 and 2.7 months in PFS (P < 0.001 and 0.01, respectively) (23).

In recurrent glioma, the initial recurrence site is frequently a few millimeters from or inside the original surgical area, infiltrates and expands around the sinus tract of the previous surgery, and is mostly distributed to satellite and multifocal lesions (24, 25), which are not easily detected under the microscope during surgery. These characteristics pose a challenge for the surgical localization of satellite and multiple tumors and complete resection. A growing number of studies have demonstrated the value of the US in locating brain tumors and assisting with their removal. Between January 2021 and September 2021, 17 patients with various brain malignancies underwent ioUS guidance, according to Giammalva et al. (20), they thought the use of ultrasound is crucial to improve surgical effectiveness and patient safety. However, few studies have reported the application of ioUS in recurrent glioma and analyzed patient outcomes. In our research, we reported ioUS localization, guided surgical resection of recurrent glioma, and detection of postoperative residual and analyzed its impact on RTV and patient outcomes.

Generally, preoperative CT and MRI will be done in the recurrent glioma surgery, but it is difficult to locate tumor tissues under the microscope because of brain shift due to the release of cerebrospinal fluid or swelling of brain tissue after opening the dura and brain shift due to tumor resection (26). In addition, the characteristics of recurrent gliomas are scattered and multiple, making it more difficult to find the tumor tissue under the microscope completely according to the surgeons’ impression. However, ioUS is not affected by brain shift (14, 27), and after opening the dura, it can still be performed to locate the tumor and guide it in real-time to find all the scattered tumor tissue, which could have assisted in achieving lesser RTV throughout. In our research, ioUS decreased the RTV (mean RTV 0.27 vs. 1.33 cm^3^, p = 0.0004) compared to patients without the guidance of ioUS. Lesser RTV was associated with a trend towards higher KPS at 6 months after the operation, lower recurrence rates, and longer recurrence intervals (median KPS 70 vs. 60, p = 0.0185; recurrence rates 71.11 vs. 91.49%, p = 0.0118; time to recurrence 7.9 vs. 6.3 months, p = 0.0013). Under previous studies (28), the outcome of patients with recurrent glioblastoma who underwent reoperations improved with decreasing postoperative RTV.

To analyze the possible reasons why intraoperative ultrasound can reduce RTV, we carefully compared the preoperative and postoperative CT, MRI, US, and pathological results of the patients, the possible reasons are as follows.

Firstly, MRI is effective in detecting intracranial lesions, but it is often incapable of differentiating between tumors, gliosis, or edema (29). Patient 1 (M.X.Y.)’s peritumoral edema may contribute to the missed diagnosis of the tumor tissue on MRI. Meanwhile, MRI is very reliable in visualizing brain tumors and residual tumors during primary surgery. However, owing to artifacts from earlier surgical therapy, its specificity is limited in recurring patients (30). MRI artifacts may also be the main cause of missed diagnosis of lesions. However, high-frequency ioUS overcomes these shortcomings and can well identify multiple lesions, thereby reducing RTV in our study.

Secondly, in term of patient 2 (Z.K.Y.), due to recurrent oligodendroglioma, which has the characteristics of scattered calcification, MRI is superior to CT in assessing tumor extent, whereas CT is most sensitive to calcification (31). MRI is not sensitive to identify calcification, and it is difficult to based on MRI to complete the resection of lesions, CT can though in preoperative identification of calcification, but can’t guide for scattered lesions during operation, and ioUS can not only locate calcifications, after removal of part of lesions in operation, under the condition of brain shift occurred, it can still guide to find other scattered lesions and achieve complete resection of multiple lesions, thereby reducing RTV in our research.

Last but not least, In the case of patient 3 (J.L.X.), the small lesion missed by MRI was only 3*3mm, recurrent gliomas with complex per cerebral structures may obscure or interfere with MRI identification of small lesions (30). On the other hand, MRI is a tomography scan, and ultrasound is a continuous, multi-directional scan, which may lead to small lesions not detected by MRI, but detected by US (32).

In our study, patients with tumors in eloquent areas in the ioUS group have less RTV and higher KPS, which might be attributed to the following factors: Firstly, we can more precisely locate the tumor using ioUS and remove it with a surgical corridor without considerably damaging the healthy functioning brain tissue around it. As a result, there is less remaining tumor and less harm. On the contrary, detecting and pinpointing the tumor without ioUS guidance may cause more harm to normal brain tissue and the boundary between the tumor and normal brain tissue cannot be properly recognized, and the doctor will decide to remove as little as possible to avoid hurting the functioning brain tissue, leaving more tumor remains. Secondly, we can enter through a surgical corridor from the surrounding non-functional cortex and utilize real-time ultrasound guidance to remove cancers below the functional cortex without harming the functional cortex while removing tumors below the functional cortex. When excision from the bypass is performed without ioUS guidance, it is difficult to detect the tumor directly, which may result in brain tissue injury during the procedure. Last but not least, the color doppler aspect of ioUS enables us to recognize blood vessels and guard against damaging the blood vessels that innervate the functioning region during tumor removal, if there is no color doppler cues, it is hard to have a solid grasp of the vascular status, and injury to arteries innervating the cortex.

According to certain research, the RTV for primary glioma surgery is decreased when using ultrasound (33). However, some studies have reported that ioUS cannot evaluate the RTV of recurrent glioma with a previous surgical cavity that hindered good contact between the brain surface and the US probe (34). To solve this problem, our experience is that during US scanning, normal saline is continuously and slowly injected into the surgical cavity, so that the probe completely fits the normal saline, without any gaps and bubbles, and the lesions can be observed.

**Figure 5:**
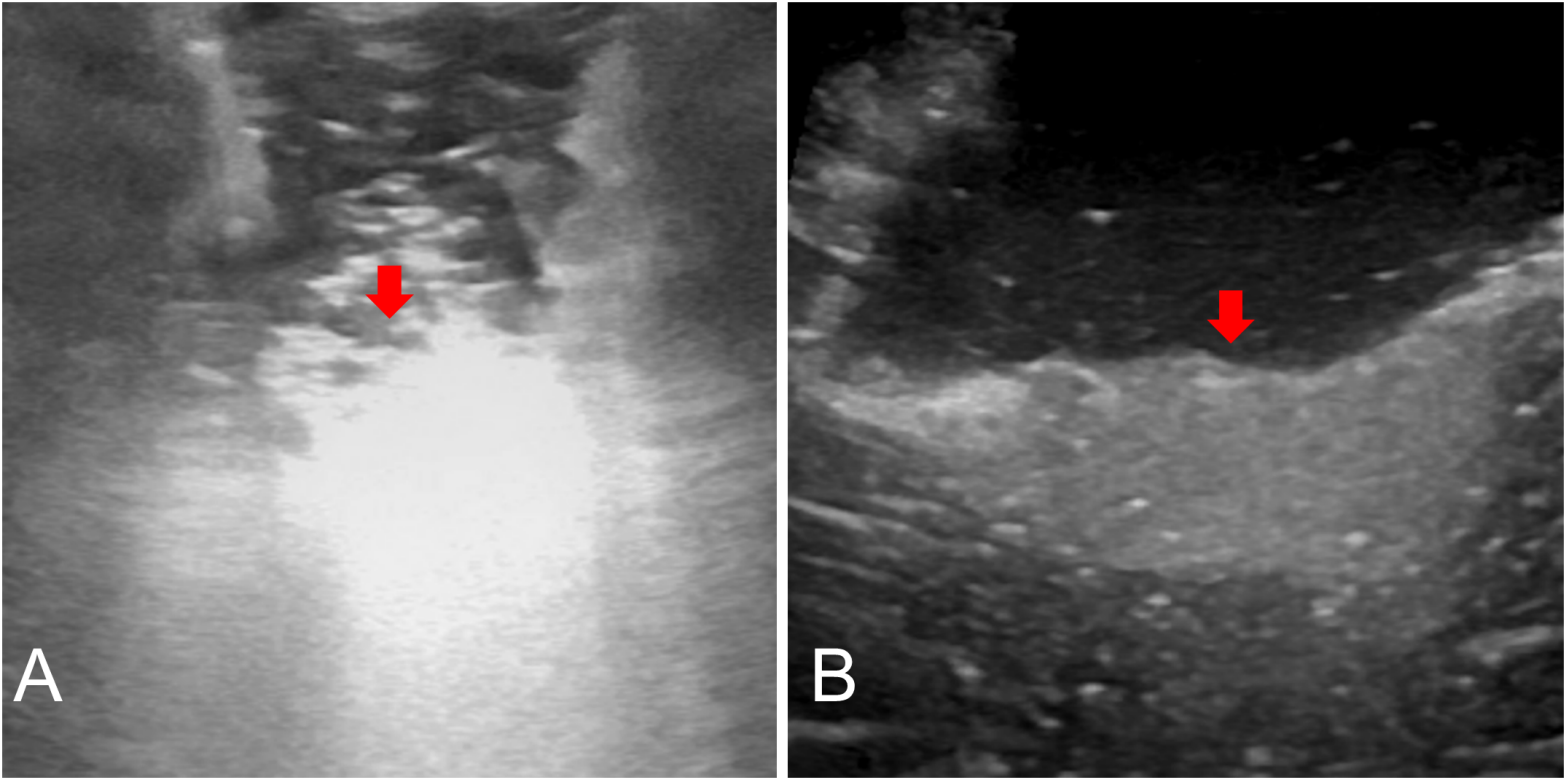
Postoperative US: Surgical cavity was covered by the hemostatic gauze (**A**, red arrow). Fully hemostatic, taking out all hemostatic materials and normal saline fills the surgical cavity, and the residual tumor can be seen (**B**, red arrow).

Nonetheless, the density of a mass and the mass differential between two neighboring tissue sections are what determine the echogenicity of the US (35). The main disadvantage is that acquiring and interpreting the US picture is doctor-dependent and subjective (36). In our study, the acquisition and interpretation of ultrasound images were done by doctors with ultrasound qualifications, which well overcame this shortcoming. Some artifacts, such as blood, air bubbles, or postoperative radiotherapy-associated alterations, confuse picture interpretation and can decrease ioUS sensitivity to detect tumor remains (37). The hyperechoic areas covered by the hemostatic gauze might have been misinterpreted as tumor infiltration and covered the underlying parenchyma, lowering the sensitivity to scan for tumor remains (**Figure 5A**). In our study, when performing a US examination of tumor residual, we achieved high-quality US images by fully hemostatic, removing all the hemostatic materials (hemostatic gauze, brain cotton, etc.) from the surgical cavity, and slowly and continuously filling the surgical cavity with normal saline to make the US probe fit perfectly with normal saline (**Figure 5B**).

IoUS was performed to identify the lesion before to dural opening and during tumor removal, as well as to detect any remaining tumor at the termination of surgery. The cerebral cortex and dura mater are extensively adherent during surgery for recurrent glioma. An wide separation, particularly in motor regions or close to significant blood vessels, can be avoided by intraoperative ultrasound localization by separating just the dura mater corresponding to the tumor from the cerebral cortex. We desire to contribute to the research of future perspectives on technology developments and potential in recurrence glioma surgery. We’d like to demonstrate how ioUS may assist surgeons in better finding tumor tissue, reducing damage to important brain tissue and blood vessels and resecting multifocal tumors properly and completely.

The limitation of our work is that it is retrospective research, and we only investigated a small number of surgeries retrospectively. We attempted to minimize retrospective bias by verifying RTV with pre- and postoperative MRI, eliminating selection bias, and avoiding grouping patients into groups based on age, tumor location, and size, or preoperative conditions. Further prospective studies such as a larger number of patients in multiple centers are required.

## Conclusion

The use of ioUS in repeat glioma surgery is feasible and worthy of being widely used clinically. It aids in achieving a lesser RTV, which improves the KPS, reduces the recurrence rate, and prolongs the time to recurrence. Despite the study’s limitations, ioUS has shown promise in terms of RTV and postoperative outcomes for the surgery of recurrent gliomas.

## Data availability statement

The original contributions presented in the study are included in the article/supplementary material. Further inquiries can be directed to the corresponding author.

## Ethics statement

The studies involving human participants were reviewed and approved by the Ethics Committee at Zhongnan Hospital of Wuhan University. Written informed consent to participate in this study was provided by the participants’ legal guardian/next of kin.

## Author contributions

Conceptualization and Supervision, JCC. Methodology, MYW, JBZ and ZYP. Data curation, JY. Writing-original draft preparation, MYW. Writing-review and editing, JY and JCC. All authors contributed to the article and approved the submitted version. 
